# Bromination of bis­(pyridin-2-yl) diselenide in methyl­ene chloride: the reaction mechanism and crystal structures of 1*H*-pyridine-2-selenenyl dibromide and its cyclo­adduct with cyclo­pentene (3a*SR*,9a*RS*)-2,3,3a,9a-tetra­hydro-1*H*-cyclo­penta­[4,5][1,3]selenazolo[3,2-*a*]pyridinium bromide

**DOI:** 10.1107/S2056989019004997

**Published:** 2019-04-25

**Authors:** Zhanna V. Matsulevich, Julia M. Lukiyanova, Vladimir I. Naumov, Galina N. Borisova, Vladimir K. Osmanov, Alexander V. Borisov, Maria M. Grishina, Victor N. Khrustalev

**Affiliations:** a R.E. Alekseev Nizhny Novgorod State Technical University, Minin St, 24, Nizhny Novgorod, 603950 , Russian Federation; b Inorganic Chemistry Department, Peoples’ Friendship University of Russia (RUDN University), 6 Miklukho-Maklay St., Moscow 117198, Russian Federation

**Keywords:** bromination, cyclo­addition reaction, bis­(pyridin-2-yl) diselenide, 2-pyridyl­selenenyl bromide, cyclo­pentene, 2,3,3a,9a-tetra­hydro-1*H*-cyclo­penta­[4,5][1,3]selenazolo[3,2-*a*]pyridinium-9 bromide, crystal structure

## Abstract

1*H*-Pyridine-2-selenenyl dibromide, C_5_H_5_NSeBr_2_, **1**, is a product of the bromination of bis­(pyridin-2-yl) diselenide in methyl­ene chloride recrystallization from methanol. Compound **1** is essentially zwitterionic: the negative charge resides on the SeBr_2_ moiety and the positive charge is delocalized over the pyridinium fragment. The C—Se distance of 1.927 (3) Å is typical of a single bond. The virtually linear Br—Se—Br moiety of 178.428 (15)° has symmetrical geometry, with Se—Br bonds of 2.5761 (4) and 2.5920 (4) Å, and is twisted by 63.79 (8)° relative to the pyridinium plane. The Se atom forms an inter­molecular Se⋯Br contact of 3.4326 (4) Å, adopting a distorted square-planar coordination. In the crystal, mol­ecules of **1** are linked by inter­molecular N—H⋯Br and C—H⋯Br hydrogen bonds, as well as by non-covalent Se⋯Br inter­actions, into a three-dimensional framework. (3a*SR*,(9a*RS*)-2,3,3a,9a-Tetra­hydro-1*H*-cyclo­penta[4,5][1,3]selenazolo[3,2-*a*]pyridinium-9 bromide, C_10_H_12_NSe^+^·Br^−^, **2**, is a product of the cyclo­addition reaction of **1** with cyclo­pentene. Compound **2** is a salt containing a selenazolopyridinium cation and a bromide anion. Both five-membered rings of the cation adopt envelope conformations. The dihedral angle between the basal planes of these rings is 62.45 (11)°. The Se atom of the cation forms two additional non-covalent inter­actions with the bromide anions at distances of 3.2715 (4) and 3.5683 (3) Å, attaining a distorted square-planar coordination. In the crystal, the cations and anions of **2** form centrosymmetric dimers by non-covalent Se⋯Br inter­actions. The dimers are linked by weak C—H⋯Br hydrogen bonds into double layers parallel to (001).

## Abstract 2

Bromination of bis­(pyridin-2-yl) diselenide in methyl­ene chloride was carried out and the mechanism is proposed. The mol­ecular and crystal structures of 1*H*-pyridine-2-selenenyl dibromide and its cyclo­adduct with cyclo­pentene were studied by X-ray diffraction.

## Chemical context   

Selenium-containing mol­ecules have attracted significant attention from chemical and medicinal scientists because of their wide range of biological activities, such as anti­tumor effects, cardiovascular protection, anti­bacterial or anti­viral effects (Banerjee & Koketsu, 2017 ▸; Zhang *et al.*, 2017 ▸; Álvarez-Pérez *et al.*, 2018 ▸; Miao *et al.*, 2018 ▸). However, the chemistry of organoselenium compounds has not been sufficiently developed in comparison with that of organosulfur compounds because of the instability of most Se-containing compounds (Ninomiya *et al.*, 2011 ▸). Thus, the synthesis, isolation and structural characterization of selenium-containing substances is essential for the further development of potential medicines.

Earlier, the product of bromination of bis­(pyridin-2-yl) diselenide in methyl­ene chloride was described by Japanese researchers (Toshimitsu *et al.*, 1984 ▸). This compound had a melting point of 388–390 K and was assigned as 2-pyridyl­selenenyl bromide based on the elemental analysis and IR spectroscopic data.
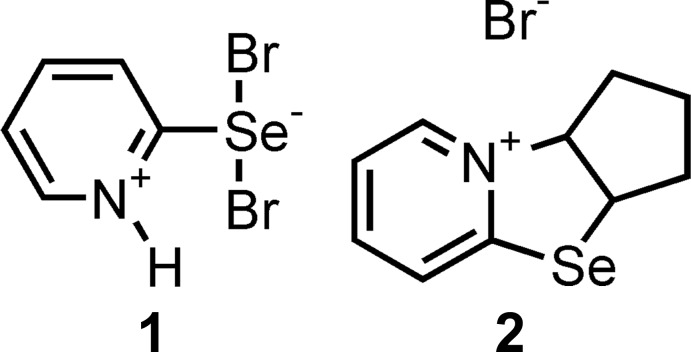


**Figure 1:**
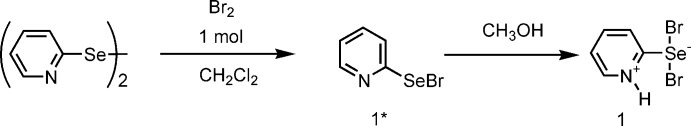
Synthesis of 1*H*-pyridine-2-selenenyl dibromide **1** by the bromination of bis­(pyridin-2-yl) diselenide in methyl­ene chloride.

However, as a result of our multiple experiments on the bromination of bis(pyridin-2-yl) diselenide under similar conditions, a product with a melting point of 373–375 K was obtained. We isolated a compound with the same melting point as that previously obtained by the Japanese authors only after recrystallization from methanol. In our opinion, it is the lower melting point product that is the 2-pyridyl­selenenyl bromide 1*. The product having a higher melting point was isolated by us and then structurally characterized by X-ray analysis to be 1*H*-pyridine-2-selenenyl dibromide **1** (Fig. 1 ▸).

**Figure 2:**
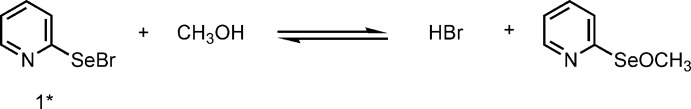
The inter­action of selenenyl bromide 1* with methanol.

**Figure 3:**
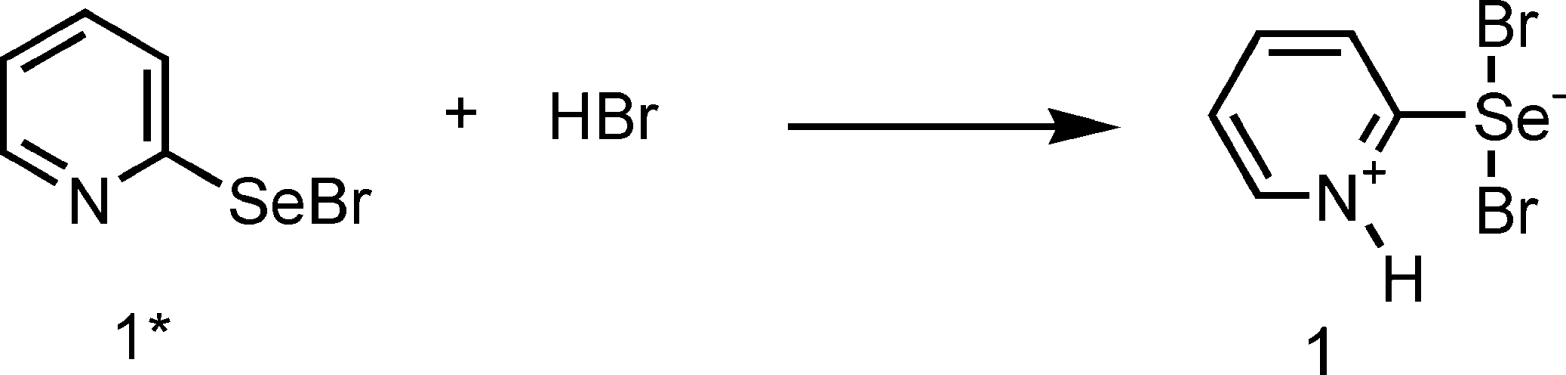
The addition reaction of hydrogen bromide to selenenyl bromide 1*.

Previously we have developed an approach to the synthesis of [1,3]thia­(selen,tellur)azolo[3,2-*a*]pyridin-4-ium derivatives *via* heterocyclization of unsaturated compounds and 2-pyridine­sulfenyl, selenenyl and tellurenyl chlorides with ring closure through the nitro­gen atom of the pyridyl fragment (Borisov *et al.*, 2010 ▸, 2012*a* ▸,*b* ▸,*c* ▸). In this case, our studies have paid particular attention to clarifying the structural characteristics of the reagents used (Borisov *et al.*, 2010 ▸; Khrustalev *et al.*, 2014 ▸, 2016 ▸). Determination of the factors providing the stability of organochalcogenyl halides is known to be an urgent challenge in general (Khrustalev *et al.*, 2014 ▸, 2016 ▸). The structural features of 2-pyridine-selenenyl and -tellurenyl chlorides have been described by us in detail (Borisov *et al.*, 2010 ▸; Khrustalev *et al.*, 2014 ▸, 2016 ▸). Moreover, we have proposed a probable mechanism of the reaction including the inter­action of selenenyl bromide 1* with methanol producing hydrogen bromide and methyl selenite (Fig. 2 ▸) (Garratt & Kabo, 1980 ▸; Reich & Jasperse, 1988 ▸). Furthermore, the subsequent addition of hydrogen bromide to selenenyl bromide 1* gives 1*H*-pyridine-2-selenenyl dibromide **1** (Fig. 3 ▸).

**Figure 4:**
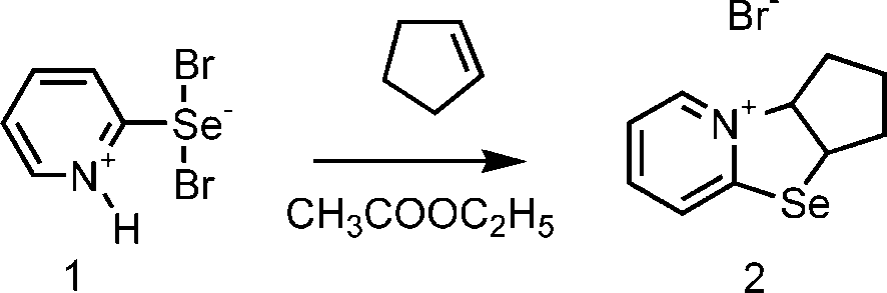
The reaction of 1*H*-pyridine-2-selenenyl dibromide **1** with cyclo­pentene.

We have also succeeded in involving 1*H*-pyridine-2-selenenyl dibromide **1** in the cyclo­addition reaction with cyclo­pentene. The product of this reaction was identified as 2,3,3a,9a-tetra­hydro-1*H*-cyclo­penta­[4,5][1,3]selenazolo[3,2-*a*]pyridinium-9 bromide (**2**) by X-ray diffraction (Fig. 4 ▸).

## Structural commentary   

**Figure 5:**
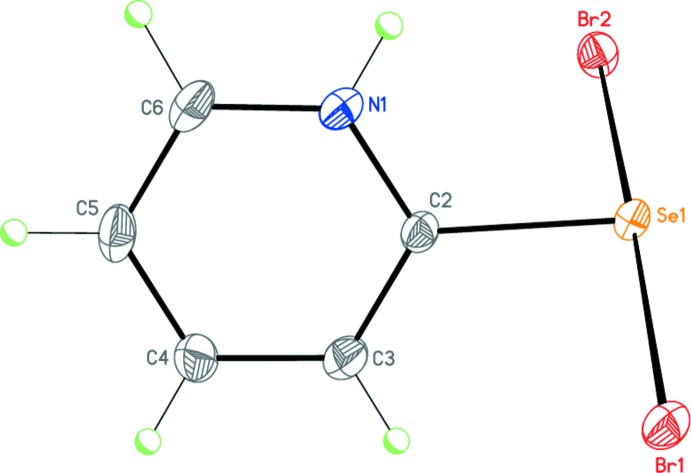
Mol­ecular structure of **1**. Displacement ellipsoids are shown at the 50% probability level. H atoms are presented as small spheres of arbitrary radius.

Compound **1**, C_5_H_5_NSeBr_2_, is essentially zwitterionic: a negative charge resides on the SeBr_2_ moiety and a positive charge is delocalized over the pyridinium fragment (Fig. 5 ▸). The C2—Se1 distance of 1.927 (3) Å is typical for a single bond [in comparison, the lengths of the C=Se bonds in related compounds are 1.817 (7) Å (Husebye *et al.*, 1997 ▸), 1.8236 (11) Å (Mammadova *et al.*, 2011 ▸) and 1.838 (2) Å (Mammadova *et al.*, 2012 ▸)]. The N1—C2 and N1—C6 bond lengths are almost equal to each other because of the aromaticity of the cyclic system. The virtually linear Br1—Se1—Br2 moiety of 178.428 (15)° has a symmetrical geometry with Se—Br bonds of 2.5761 (4) and 2.5920 (4) Å and is twisted by 63.79 (8)° relative to the pyridinium plane. The slight elongation of the Se1—Br2 bond in comparison with the Se1—Br1 bond is explained by the involvement of the Br2 atom in the inter­molecular secondary Se1⋯Br2(*x*, 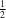 − *y*, 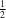 + *z*) inter­action [3.4326 (4) Å]. Thus, the selenium atom adopts a distorted square-planar coordination.

**Figure 6:**
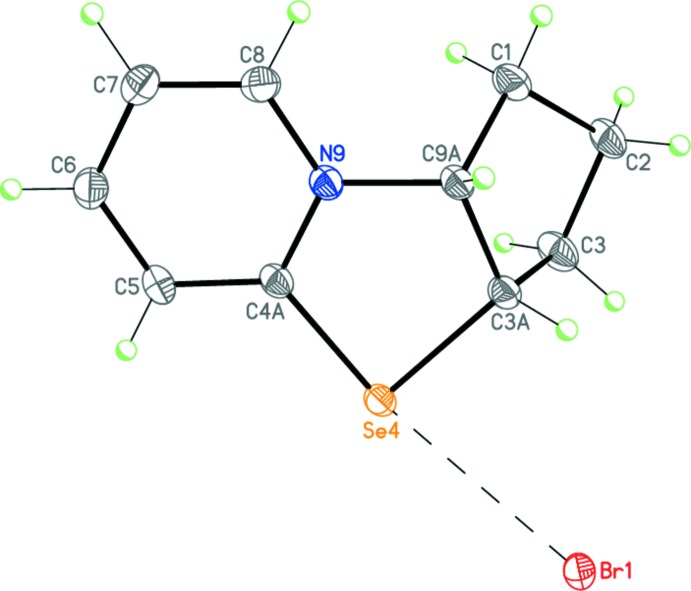
Mol­ecular structure of **2**. Displacement ellipsoids are shown at the 50% probability level. H atoms are presented as small spheres of arbitrary radius. The dashed line indicates the inter­molecular non-covalent Se⋯Br inter­action.

Compound **2**, C_10_H_12_NSeBr, is a salt containing a selenazolopyridinium cation and a bromide anion (Fig. 6 ▸). The five-membered heterocycle of the cation adopts a flattened envelope conformation with the C3*A* carbon atom deviating by 0.274 (3) Å from the plane through the other ring atoms. The cyclo­pentane fragment has the usual envelope conformation with the C2 carbon atom deviating from the plane through the other ring atoms by 0.648 (4) Å. The dihedral angle between the basal planes of the two five-membered rings of the cation is 62.45 (11)°. The selenium atom of the cation forms two additional non-covalent inter­actions with the bromide anions at distances of 3.2715 (4) Å [Se4⋯Br1(*x*, 1 + *y*, *z*)] and 3.5683 (3) Å [Se4⋯Br1(1–*x*, 1–*y*, –*z*)], affording a distorted square-planar coordination.

Cation **2** has two asymmetric C3*A* and C9*A* carbon atoms. The crystal of the compound is racemic with the following relative configurations of the centers – *rac*-3a*SR*,9a*RS*.

## Supra­molecular features   

**Figure 7:**
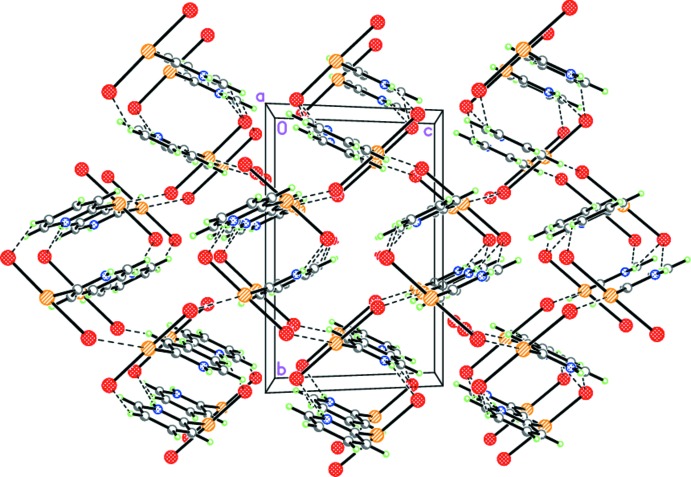
Crystal structure of **1** viewed along the ***a*** axis. Dashed lines indicate the inter­molecular N—H⋯Br and C—H⋯Br hydrogen bonds as well as the non-covalent Se⋯Br inter­actions.

In the crystal of **1**, mol­ecules are linked by inter­molecular N—H⋯Br and C—H⋯Br hydrogen bonds (Table 1 ▸) as well as by the non-covalent Se⋯Br inter­actions (see above) into a three-dimensional framework (Fig. 7 ▸).

**Figure 8:**
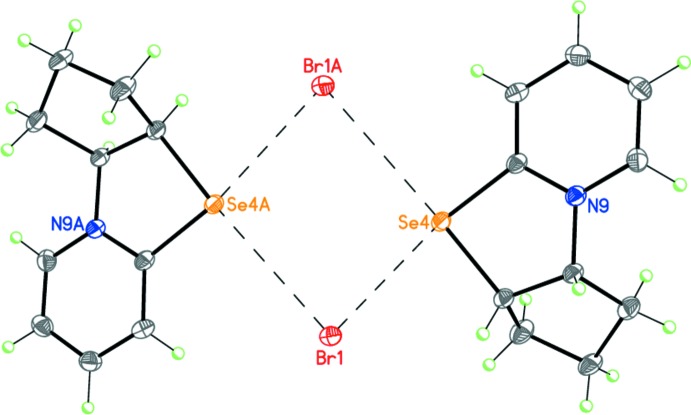
Dimeric structure of **2**. Dashed lines indicate the inter­molecular non-covalent Se⋯Br inter­actions. **[Symmetry code: (A) ???]**

**Figure 9:**
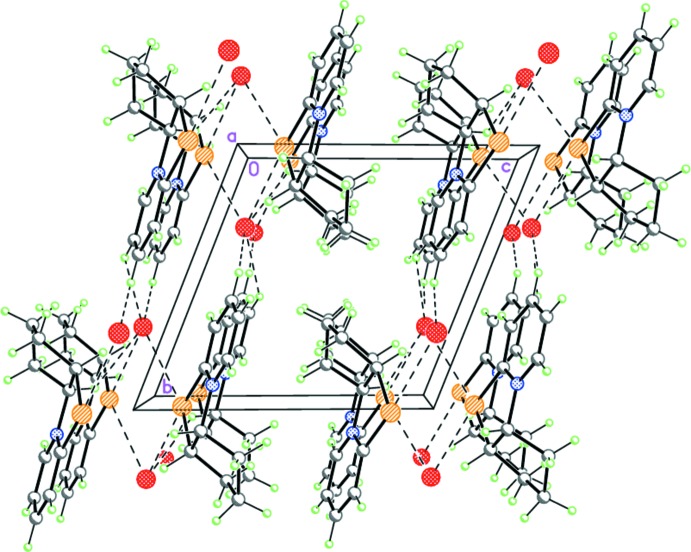
Crystal structure of **2** showing the double layers parallel to (001). Dashed lines indicate the inter­molecular C—H⋯Br hydrogen bonds as well as the non-covalent Se⋯Br inter­actions.

In the crystal of **2**, the cations and anions are linked by Se⋯Br inter­actions, forming centrosymmetric dimers (Fig. 8 ▸). The dimers are linked by weak C—H⋯Br hydrogen bonds (Table 2 ▸) into double layers parallel to (001) (Fig. 9 ▸).

## Database survey   

A search of the Cambridge Structural Database (CSD, Version 5.40; Groom *et al.*, 2016 ▸) for zwitterionic mol­ecules containing the T-shaped SeBr_2_ fragment yielded 22 such compounds. In 16 of them, the hypervalent SeBr_2_ fragments have asymmetric geometries, with the difference in the two Se—Br bond lengths more than or close to 0.1 Å, which is explained by inter­molecular non-covalent inter­actions in the crystals. Moreover, 12 out of these 16 crystal structures revealed the presence of inter­molecular non-covalent Se⋯Br inter­actions with distances of 3.3374 (5)–3.556 (1) Å.

Remarkably, the inter­molecular non-covalent Se⋯Br inter­action of 3.2715 (4) Å observed in the crystal of **2** is the strongest one found in the compounds of this type – between the diorganyl selenide unit and the bromide anion.

## Synthesis and crystallization   

2-Pyridine­selenenyl bromide (1*). A solution of bromine (0.32 g, 2 mmol) in ethyl­ene chloride (10 ml) was added to a solution of bis­(pyridin-2-yl)diselenide (0.628 g, 2 mmol) in methyl­ene chloride (10 ml) at room temperature. After 30 min, the solvent was removed under vacuum. The residue was washed with diethyl ether. Yield 0.93 g (98%), bright-yellow powder, m.p. 373–375 K. Analysis calculated for C_5_H_4_BrNSe (%): C, 25.35; H, 1.70; N, 5.91. Found (%): C, 25.31; H, 1.68; N, 5.89.

**1** ***H*** **-Pyridine-2-selenenyl dibromide (1).** Compound 1* was recrystallized from methanol. Yield 0.59 g (92%), orange crystals, m.p. 388–390 K. Analysis calculated for C_5_H_5_Br_2_NSe (%): C, 18.89; H, 1.59; N, 4.41. Found (%): C, 18.81; H, 1.55; N, 4.37.

**2,3,3a,9a-Tetra­hydro-1** ***H*** **-cyclo­penta­[4,5][1,3]σelenazolo[3,2-a]pyridinium-9 bromide (2)**. A solution of cyclo­pentene (0.034 g, 0.5 mmol) in ethyl acetate (5 ml) was added to a solution of **1** (0.159 g, 0.5 mmol) in ethyl acetate (10 ml) at room temperature. The reaction mixture was kept at room temperature for 24 h, then the solvent was removed under vacuum. The crude white solid was recrystallized from methyl­ene chloride. Single crystals suitable for X-ray diffraction analysis were obtained by recrystallization from methylene chloride. Yield 0.133 g (87%), white powder, m.p. 463–465 K. Analysis calculated for C_10_H_12_BrNSe (%): C, 39.29; H, 3.91; N, 4.52. Found (%): C, 39.38; H, 3.97; N, 4.59. ^1^H NMR (DMSO-*d*_6_, 400 MHz, 302 K): *δ* = 8.98 (*d*, 1H, H8, *J* = 6.3 Hz), 8.20 (*m*, 2H, H5, H6), 7.74 (*ddd*, 1H, H7, *J* = 8.9 Hz, *J* = 5.8 Hz, *J* = 3.2 Hz), 5.78 (*td*, 1H, H9a, *J* = 8.4 Hz, *J* = 3.9 Hz), 4.66 (*m*, 1H, H3a), 2.34, 2.09, 1.71 (*m*, 6H, 3CH_2_).

## Refinement   

Crystal data, data collection and structure refinement details are summarized in Table 3 ▸. The hydrogen atom of the NH-group in **1** was localized in the difference-Fourier map and refined isotropically with fixed displacement parameters [*U*_iso_(H) = 1.2*U*_eq_(N)]. The other hydrogen atoms in **1** and **2** were placed in calculated positions with C—H = 0.95–1.00 Å and refined using a riding model with fixed isotropic displacement parameters [*U*_iso_(H) = 1.5*U*_eq_(C) for the CH_3_-groups and 1.2*U*_eq_(C) for the other groups].

## Supplementary Material

Crystal structure: contains datablock(s) global, 1, 2. DOI: 10.1107/S2056989019004997/yk2121sup1.cif

Structure factors: contains datablock(s) 1. DOI: 10.1107/S2056989019004997/yk21211sup2.hkl

Structure factors: contains datablock(s) 2. DOI: 10.1107/S2056989019004997/yk21212sup3.hkl

CCDC references: 1909603, 1909602

Additional supporting information:  crystallographic information; 3D view; checkCIF report

## Figures and Tables

**Table 1:** Hydrogen-bond geometry (Å, °) for **1** [Chem scheme1]

*D*—H⋯*A*	*D*—H	H⋯*A*	*D*⋯*A*	*D*—H⋯*A*
N1—H1⋯Br1^i^	0.86 (4)	2.50 (4)	3.305 (3)	156 (3)
C5—H5⋯Br1^ii^	0.95	2.92	3.790 (3)	153

Symmetry codes: (i) 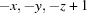; (ii) 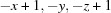.

**Table 2:** Hydrogen-bond geometry (Å, °) for **2** [Chem scheme1]

*D*—H⋯*A*	*D*—H	H⋯*A*	*D*⋯*A*	*D*—H⋯*A*
C7—H7⋯Br1^i^	0.95	2.91	3.728 (2)	145
C9*A*—H9*A*⋯Br1^ii^	1.00	2.82	3.614 (2)	137

Symmetry codes: (i) 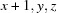; (ii) 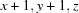.

**Table 3:** Experimental details

	**1**	**2**
Crystal data		
Chemical formula	C_5_H_5_Br_2_NSe	C_10_H_12_NSe^+^·Br^−^
*M*_r_	317.86	305.07
Crystal system, space group	Monoclinic, *P*2_1_/*c*	Triclinic, *P* 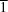
Temperature (K)	120	120
*a*, *b*, *c* (Å)	8.0971 (6), 12.6116 (10), 8.7325 (7)	6.3333 (5), 9.0515 (7), 9.5807 (7)
α, β, γ (°)	90, 114.975 (1), 90	111.350 (1), 93.657 (2), 93.543 (1)
*V* (Å^3^)	808.36 (11)	508.35 (7)
*Z*	4	2
Radiation type	Mo *K*α	Mo *K*α
μ (mm^−1^)	14.44	7.57
Crystal size (mm)	0.20 × 0.20 × 0.15	0.30 × 0.20 × 0.20

Data collection		
Diffractometer	Bruker APEXII CCD	Bruker APEXII CCD
Absorption correction	Multi-scan (*SADABS*; Sheldrick, 2003 ▸)	Multi-scan (*SADABS*; Sheldrick, 2003 ▸)
*T*_min_, *T*_max_	0.063, 0.104	0.115, 0.154
No. of measured, independent and observed [*I* > 2σ(*I*)] reflections	12425, 2959, 2426	7982, 3711, 3156
*R*_int_	0.051	0.029
(sin θ/λ)_max_ (Å^−1^)	0.759	0.760

Refinement		
*R*[*F* ^2^ > 2σ(*F* ^2^)], *wR*(*F* ^2^), *S*	0.032, 0.074, 1.03	0.030, 0.080, 1.06
No. of reflections	2959	3711
No. of parameters	85	118
H-atom treatment	H atoms treated by a mixture of independent and constrained refinement	H-atom parameters constrained
Δρ_max_, Δρ_min_ (e Å^−3^)	1.37, −1.06	0.64, −1.05

Computer programs: *APEX2* (Bruker, 2005 ▸), *SAINT* (Bruker, 2002 ▸) and *SHELXTL* (Sheldrick, 2008 ▸).

## supplementary crystallographic information

### Dibromo(pyridin-1-ium-2-yl)selanide (1) Crystal data

**Table:**

C_5_H_5_Br_2_NSe	*F*(000) = 584
*M**_r_* = 317.86	*D*_x_ = 2.612 Mg m^−^^3^
Monoclinic, *P*2_1_/*c*	Mo *K*α radiation, λ = 0.71073 Å
*a* = 8.0971 (6) Å	Cell parameters from 4271 reflections
*b* = 12.6116 (10) Å	θ = 2.8–32.6°
*c* = 8.7325 (7) Å	µ = 14.44 mm^−^^1^
β = 114.975 (1)°	*T* = 120 K
*V* = 808.36 (11) Å^3^	Prism, orange
*Z* = 4	0.20 × 0.20 × 0.15 mm

### Dibromo(pyridin-1-ium-2-yl)selanide (1) Data collection

**Table:**

Bruker APEXII CCD diffractometer	2426 reflections with *I* > 2σ(*I*)
Radiation source: fine-focus sealed tube	*R*_int_ = 0.051
φ and ω scans	θ_max_ = 32.6°, θ_min_ = 2.8°
Absorption correction: multi-scan (SADABS; Sheldrick, 2003)	*h* = −12→12
*T*_min_ = 0.063, *T*_max_ = 0.104	*k* = −18→19
12425 measured reflections	*l* = −13→13
2959 independent reflections	

### Dibromo(pyridin-1-ium-2-yl)selanide (1) Refinement

**Table:**

Refinement on *F*^2^	Primary atom site location: difference Fourier map
Least-squares matrix: full	Secondary atom site location: difference Fourier map
*R*[*F*^2^ > 2σ(*F*^2^)] = 0.032	Hydrogen site location: mixed
*wR*(*F*^2^) = 0.074	H atoms treated by a mixture of independent and constrained refinement
*S* = 1.03	*w* = 1/[σ^2^(*F*_o_^2^) + (0.0302*P*)^2^ + 0.5977*P*] where *P* = (*F*_o_^2^ + 2*F*_c_^2^)/3
2959 reflections	(Δ/σ)_max_ = 0.001
85 parameters	Δρ_max_ = 1.37 e Å^−^^3^
0 restraints	Δρ_min_ = −1.06 e Å^−^^3^

### Dibromo(pyridin-1-ium-2-yl)selanide (1) Special details

**Table:**

Geometry. All esds (except the esd in the dihedral angle between two l.s. planes) are estimated using the full covariance matrix. The cell esds are taken into account individually in the estimation of esds in distances, angles and torsion angles; correlations between esds in cell parameters are only used when they are defined by crystal symmetry. An approximate (isotropic) treatment of cell esds is used for estimating esds involving l.s. planes.

### Dibromo(pyridin-1-ium-2-yl)selanide (1) Fractional atomic coordinates and isotropic or equivalent isotropic displacement parameters (Å^2^)

**Table:**

	*x*	*y*	*z*	*U*_iso_*/*U*_eq_	
Br1	0.26838 (4)	0.03212 (2)	0.84166 (3)	0.01817 (8)	
Br2	−0.14496 (4)	0.29163 (3)	0.38147 (4)	0.01989 (8)	
Se1	0.06027 (4)	0.16227 (2)	0.61494 (3)	0.01433 (7)	
N1	0.1263 (4)	0.0928 (2)	0.3377 (3)	0.0161 (5)	
H1	0.012 (6)	0.079 (3)	0.298 (5)	0.019*	
C2	0.2053 (4)	0.1389 (2)	0.4909 (4)	0.0143 (5)	
C3	0.3877 (4)	0.1667 (3)	0.5524 (4)	0.0205 (6)	
H3	0.4467	0.1997	0.6598	0.025*	
C4	0.4837 (4)	0.1459 (3)	0.4561 (4)	0.0214 (6)	
H4	0.6089	0.1643	0.4981	0.026*	
C5	0.3971 (5)	0.0985 (3)	0.2989 (4)	0.0218 (6)	
H5	0.4616	0.0839	0.2323	0.026*	
C6	0.2157 (4)	0.0729 (3)	0.2415 (4)	0.0200 (6)	
H6	0.1533	0.0412	0.1335	0.024*	

### Dibromo(pyridin-1-ium-2-yl)selanide (1) Atomic displacement parameters (Å^2^)

**Table:**

	*U*^11^	*U*^22^	*U*^33^	*U*^12^	*U*^13^	*U*^23^
Br1	0.01837 (14)	0.02401 (16)	0.01174 (12)	0.00296 (10)	0.00598 (10)	0.00250 (10)
Br2	0.01919 (14)	0.02324 (16)	0.01808 (14)	0.00366 (11)	0.00869 (11)	0.00614 (11)
Se1	0.01580 (13)	0.01724 (14)	0.01117 (12)	−0.00047 (10)	0.00690 (10)	−0.00082 (9)
N1	0.0186 (11)	0.0173 (12)	0.0115 (10)	−0.0010 (9)	0.0056 (9)	−0.0011 (9)
C2	0.0159 (12)	0.0165 (13)	0.0112 (11)	0.0006 (10)	0.0065 (10)	0.0012 (10)
C3	0.0202 (14)	0.0254 (16)	0.0157 (13)	−0.0041 (11)	0.0075 (11)	−0.0051 (11)
C4	0.0194 (14)	0.0283 (17)	0.0186 (14)	0.0005 (12)	0.0102 (12)	0.0028 (12)
C5	0.0270 (15)	0.0257 (16)	0.0187 (14)	0.0070 (12)	0.0155 (12)	0.0037 (12)
C6	0.0276 (14)	0.0204 (15)	0.0123 (12)	0.0038 (12)	0.0087 (11)	−0.0004 (10)

### Dibromo(pyridin-1-ium-2-yl)selanide (1) Geometric parameters (Å, º)

**Table:**

Br1—Se1	2.5761 (4)	C3—C4	1.390 (4)
Br2—Se1	2.5920 (4)	C3—H3	0.9500
Se1—C2	1.927 (3)	C4—C5	1.386 (5)
N1—C6	1.344 (4)	C4—H4	0.9500
N1—C2	1.347 (4)	C5—C6	1.375 (5)
N1—H1	0.86 (4)	C5—H5	0.9500
C2—C3	1.387 (4)	C6—H6	0.9500
			
C2—Se1—Br1	88.76 (9)	C4—C3—H3	120.2
C2—Se1—Br2	89.69 (8)	C5—C4—C3	120.1 (3)
Br1—Se1—Br2	178.428 (15)	C5—C4—H4	119.9
C6—N1—C2	123.1 (3)	C3—C4—H4	119.9
C6—N1—H1	119 (3)	C6—C5—C4	118.6 (3)
C2—N1—H1	118 (3)	C6—C5—H5	120.7
N1—C2—C3	118.4 (3)	C4—C5—H5	120.7
N1—C2—Se1	118.5 (2)	N1—C6—C5	120.2 (3)
C3—C2—Se1	123.1 (2)	N1—C6—H6	119.9
C2—C3—C4	119.6 (3)	C5—C6—H6	119.9
C2—C3—H3	120.2		
			
C6—N1—C2—C3	−0.3 (4)	C2—C3—C4—C5	0.5 (5)
C6—N1—C2—Se1	−179.9 (2)	C3—C4—C5—C6	0.1 (5)
N1—C2—C3—C4	−0.4 (5)	C2—N1—C6—C5	0.9 (5)
Se1—C2—C3—C4	179.2 (2)	C4—C5—C6—N1	−0.8 (5)

### Dibromo(pyridin-1-ium-2-yl)selanide (1) Hydrogen-bond geometry (Å, º)

**Table:**

*D*—H···*A*	*D*—H	H···*A*	*D*···*A*	*D*—H···*A*
N1—H1···Br1^i^	0.86 (4)	2.50 (4)	3.305 (3)	156 (3)
C5—H5···Br1^ii^	0.95	2.92	3.790 (3)	153

Symmetry codes: (i) −*x*, −*y*, −*z*+1; (ii) −*x*+1, −*y*, −*z*+1.

### 7-Selena-1λ5-azatricyclo[6.4.0.02,6]dodeca-1(12),8,10-trien-1-ylium bromide (2) Crystal data

**Table:**

C_10_H_12_NSe^+^·Br^−^	*Z* = 2
*M**_r_* = 305.07	*F*(000) = 296
Triclinic, *P*1	*D*_x_ = 1.993 Mg m^−^^3^
*a* = 6.3333 (5) Å	Mo *K*α radiation, λ = 0.71073 Å
*b* = 9.0515 (7) Å	Cell parameters from 4390 reflections
*c* = 9.5807 (7) Å	θ = 2.3–32.7°
α = 111.350 (1)°	µ = 7.57 mm^−^^1^
β = 93.657 (2)°	*T* = 120 K
γ = 93.543 (1)°	Prism, colourless
*V* = 508.35 (7) Å^3^	0.30 × 0.20 × 0.20 mm

### 7-Selena-1λ5-azatricyclo[6.4.0.02,6]dodeca-1(12),8,10-trien-1-ylium bromide (2) Data collection

**Table:**

Bruker APEXII CCD diffractometer	3156 reflections with *I* > 2σ(*I*)
Radiation source: fine-focus sealed tube	*R*_int_ = 0.029
φ and ω scans	θ_max_ = 32.7°, θ_min_ = 2.3°
Absorption correction: multi-scan (SADABS; Sheldrick, 2003)	*h* = −9→9
*T*_min_ = 0.115, *T*_max_ = 0.154	*k* = −13→13
7982 measured reflections	*l* = −14→14
3711 independent reflections	

### 7-Selena-1λ5-azatricyclo[6.4.0.02,6]dodeca-1(12),8,10-trien-1-ylium bromide (2) Refinement

**Table:**

Refinement on *F*^2^	Primary atom site location: difference Fourier map
Least-squares matrix: full	Secondary atom site location: difference Fourier map
*R*[*F*^2^ > 2σ(*F*^2^)] = 0.030	Hydrogen site location: inferred from neighbouring sites
*wR*(*F*^2^) = 0.080	H-atom parameters constrained
*S* = 1.06	*w* = 1/[σ^2^(*F*_o_^2^) + (0.0431*P*)^2^ + 0.1817*P*] where *P* = (*F*_o_^2^ + 2*F*_c_^2^)/3
3711 reflections	(Δ/σ)_max_ = 0.001
118 parameters	Δρ_max_ = 0.64 e Å^−^^3^
0 restraints	Δρ_min_ = −1.05 e Å^−^^3^

### 7-Selena-1λ5-azatricyclo[6.4.0.02,6]dodeca-1(12),8,10-trien-1-ylium bromide (2) Special details

**Table:**

Geometry. All esds (except the esd in the dihedral angle between two l.s. planes) are estimated using the full covariance matrix. The cell esds are taken into account individually in the estimation of esds in distances, angles and torsion angles; correlations between esds in cell parameters are only used when they are defined by crystal symmetry. An approximate (isotropic) treatment of cell esds is used for estimating esds involving l.s. planes.

### 7-Selena-1λ5-azatricyclo[6.4.0.02,6]dodeca-1(12),8,10-trien-1-ylium bromide (2) Fractional atomic coordinates and isotropic or equivalent isotropic displacement parameters (Å^2^)

**Table:**

	*x*	*y*	*z*	*U*_iso_*/*U*_eq_	
Br1	0.57452 (3)	0.30220 (3)	0.11384 (2)	0.01988 (7)	
C1	1.3458 (4)	1.1581 (3)	0.4167 (3)	0.0220 (4)	
H1A	1.3149	1.1019	0.4855	0.026*	
H1B	1.5016	1.1718	0.4139	0.026*	
C2	1.2526 (4)	1.3189 (3)	0.4669 (3)	0.0227 (4)	
H2A	1.3228	1.3911	0.4237	0.027*	
H2B	1.2658	1.3710	0.5780	0.027*	
C3	1.0180 (4)	1.2722 (3)	0.4033 (3)	0.0243 (5)	
H3A	0.9389	1.2208	0.4619	0.029*	
H3B	0.9463	1.3656	0.4012	0.029*	
C3A	1.0408 (3)	1.1551 (3)	0.2450 (2)	0.0166 (4)	
H3C	1.0710	1.2169	0.1796	0.020*	
Se4	0.79630 (3)	0.99892 (2)	0.14860 (2)	0.01599 (6)	
C4A	0.9599 (3)	0.8444 (3)	0.1826 (2)	0.0162 (4)	
C5	0.8875 (4)	0.6872 (3)	0.1569 (3)	0.0193 (4)	
H5	0.7437	0.6487	0.1212	0.023*	
C6	1.0290 (4)	0.5881 (3)	0.1845 (3)	0.0213 (4)	
H6	0.9816	0.4813	0.1696	0.026*	
C7	1.2422 (4)	0.6449 (3)	0.2342 (3)	0.0212 (4)	
H7	1.3406	0.5773	0.2520	0.025*	
C8	1.3054 (3)	0.7997 (3)	0.2566 (3)	0.0191 (4)	
H8	1.4499	0.8392	0.2880	0.023*	
N9	1.1641 (3)	0.8974 (2)	0.2342 (2)	0.0158 (3)	
C9A	1.2350 (3)	1.0656 (3)	0.2576 (2)	0.0163 (4)	
H9A	1.3328	1.0679	0.1803	0.020*	

### 7-Selena-1λ5-azatricyclo[6.4.0.02,6]dodeca-1(12),8,10-trien-1-ylium bromide (2) Atomic displacement parameters (Å^2^)

**Table:**

	*U*^11^	*U*^22^	*U*^33^	*U*^12^	*U*^13^	*U*^23^
Br1	0.01950 (11)	0.01593 (11)	0.02337 (11)	−0.00059 (8)	−0.00022 (8)	0.00698 (8)
C1	0.0221 (10)	0.0198 (10)	0.0187 (10)	−0.0016 (8)	−0.0044 (8)	0.0022 (8)
C2	0.0232 (10)	0.0180 (10)	0.0197 (10)	−0.0035 (8)	−0.0023 (8)	−0.0001 (8)
C3	0.0215 (10)	0.0216 (11)	0.0208 (10)	0.0006 (8)	0.0003 (8)	−0.0023 (8)
C3A	0.0171 (9)	0.0145 (9)	0.0154 (8)	0.0003 (7)	−0.0011 (7)	0.0026 (7)
Se4	0.01420 (10)	0.01455 (10)	0.01694 (10)	0.00013 (7)	−0.00074 (7)	0.00368 (8)
C4A	0.0154 (8)	0.0153 (9)	0.0145 (8)	0.0005 (7)	0.0009 (7)	0.0018 (7)
C5	0.0193 (9)	0.0157 (9)	0.0195 (9)	−0.0025 (7)	0.0007 (7)	0.0034 (8)
C6	0.0258 (10)	0.0166 (10)	0.0209 (10)	0.0007 (8)	0.0013 (8)	0.0066 (8)
C7	0.0238 (10)	0.0191 (10)	0.0222 (10)	0.0060 (8)	0.0029 (8)	0.0083 (8)
C8	0.0181 (9)	0.0204 (10)	0.0183 (9)	0.0034 (8)	0.0003 (7)	0.0066 (8)
N9	0.0156 (7)	0.0147 (8)	0.0153 (7)	0.0004 (6)	0.0007 (6)	0.0039 (6)
C9A	0.0156 (8)	0.0147 (9)	0.0158 (8)	−0.0009 (7)	−0.0005 (7)	0.0031 (7)

### 7-Selena-1λ5-azatricyclo[6.4.0.02,6]dodeca-1(12),8,10-trien-1-ylium bromide (2) Geometric parameters (Å, º)

**Table:**

C1—C2	1.527 (3)	Se4—C4A	1.898 (2)
C1—C9A	1.542 (3)	C4A—N9	1.347 (3)
C1—H1A	0.9900	C4A—C5	1.396 (3)
C1—H1B	0.9900	C5—C6	1.385 (3)
C2—C3	1.542 (3)	C5—H5	0.9500
C2—H2A	0.9900	C6—C7	1.403 (3)
C2—H2B	0.9900	C6—H6	0.9500
C3—C3A	1.523 (3)	C7—C8	1.367 (3)
C3—H3A	0.9900	C7—H7	0.9500
C3—H3B	0.9900	C8—N9	1.356 (3)
C3A—C9A	1.536 (3)	C8—H8	0.9500
C3A—Se4	1.961 (2)	N9—C9A	1.492 (3)
C3A—H3C	1.0000	C9A—H9A	1.0000
			
C2—C1—C9A	104.40 (19)	N9—C4A—C5	119.8 (2)
C2—C1—H1A	110.9	N9—C4A—Se4	114.19 (16)
C9A—C1—H1A	110.9	C5—C4A—Se4	125.99 (16)
C2—C1—H1B	110.9	C6—C5—C4A	118.9 (2)
C9A—C1—H1B	110.9	C6—C5—H5	120.6
H1A—C1—H1B	108.9	C4A—C5—H5	120.6
C1—C2—C3	102.42 (19)	C5—C6—C7	120.2 (2)
C1—C2—H2A	111.3	C5—C6—H6	119.9
C3—C2—H2A	111.3	C7—C6—H6	119.9
C1—C2—H2B	111.3	C8—C7—C6	118.7 (2)
C3—C2—H2B	111.3	C8—C7—H7	120.7
H2A—C2—H2B	109.2	C6—C7—H7	120.7
C3A—C3—C2	101.23 (18)	N9—C8—C7	120.7 (2)
C3A—C3—H3A	111.5	N9—C8—H8	119.6
C2—C3—H3A	111.5	C7—C8—H8	119.6
C3A—C3—H3B	111.5	C4A—N9—C8	121.68 (19)
C2—C3—H3B	111.5	C4A—N9—C9A	117.87 (18)
H3A—C3—H3B	109.3	C8—N9—C9A	120.34 (18)
C3—C3A—C9A	106.34 (17)	N9—C9A—C3A	109.62 (17)
C3—C3A—Se4	115.96 (15)	N9—C9A—C1	112.33 (18)
C9A—C3A—Se4	108.73 (14)	C3A—C9A—C1	105.39 (17)
C3—C3A—H3C	108.5	N9—C9A—H9A	109.8
C9A—C3A—H3C	108.5	C3A—C9A—H9A	109.8
Se4—C3A—H3C	108.5	C1—C9A—H9A	109.8
C4A—Se4—C3A	87.21 (9)		
			
C9A—C1—C2—C3	38.3 (2)	Se4—C4A—N9—C9A	−0.1 (2)
C1—C2—C3—C3A	−44.9 (2)	C7—C8—N9—C4A	−3.3 (3)
C2—C3—C3A—C9A	34.7 (2)	C7—C8—N9—C9A	−179.5 (2)
C2—C3—C3A—Se4	155.69 (16)	C4A—N9—C9A—C3A	11.0 (2)
C3A—Se4—C4A—N9	−7.96 (16)	C8—N9—C9A—C3A	−172.69 (19)
C3A—Se4—C4A—C5	172.9 (2)	C4A—N9—C9A—C1	127.8 (2)
N9—C4A—C5—C6	−0.5 (3)	C8—N9—C9A—C1	−55.9 (3)
Se4—C4A—C5—C6	178.57 (17)	C3—C3A—C9A—N9	109.6 (2)
C4A—C5—C6—C7	−1.3 (3)	Se4—C3A—C9A—N9	−15.9 (2)
C5—C6—C7—C8	0.9 (3)	C3—C3A—C9A—C1	−11.5 (2)
C6—C7—C8—N9	1.4 (3)	Se4—C3A—C9A—C1	−137.02 (15)
C5—C4A—N9—C8	2.8 (3)	C2—C1—C9A—N9	−136.01 (19)
Se4—C4A—N9—C8	−176.36 (16)	C2—C1—C9A—C3A	−16.7 (2)
C5—C4A—N9—C9A	179.04 (19)		

### 7-Selena-1λ5-azatricyclo[6.4.0.02,6]dodeca-1(12),8,10-trien-1-ylium bromide (2) Hydrogen-bond geometry (Å, º)

**Table:**

*D*—H···*A*	*D*—H	H···*A*	*D*···*A*	*D*—H···*A*
C7—H7···Br1^i^	0.95	2.91	3.728 (2)	145
C9*A*—H9*A*···Br1^ii^	1.00	2.82	3.614 (2)	137

Symmetry codes: (i) *x*+1, *y*, *z*; (ii) *x*+1, *y*+1, *z*.
